# Pharmacodynamic modeling of moderate sedation and rationale for dosing using midazolam, propofol and alfentanil

**DOI:** 10.1186/s40360-023-00642-5

**Published:** 2023-01-16

**Authors:** Jing-Yang Liou, I-Ting Kuo, Weng-Kuei Chang, Chien-Kun Ting, Mei-Yung Tsou

**Affiliations:** 1 Department of Anesthesia, Taipei Veterans General Hospital, Taipei Veterans General Hospital, No.201, Sec. 2, Shipai Rd., Beitou District, 11217 Taipei, Taiwan; 2 Department of Biomedical Engineering, National Yang-Ming Chiao Tung University, Taipei, Taiwan; 3 School of Medicine, National Yang-Ming Chiao Tung University, Taipei, Taiwan; 4 Institute of Emergency and Critical Care Medicine, National Yang Ming Chiao Tung University, Taipei, Taiwan

**Keywords:** Endoscopy, Moderate sedation, Pharmacodynamics, Computer Simulation

## Abstract

**Purpose:**

Regulations have broadened to allow moderate sedation administration for gastrointestinal endoscopy by non-anesthesia personnel. The line between moderate and deep sedation is ambiguous. Deep sedation offers patient comfort as well as greater safety concerns. Unintended deep sedation can occur if drug interactions are overlooked. We present a pharmacodynamic model for moderate sedation using midazolam, alfentanil and propofol. The model is suitable for training and devising rationales for appropriate dosing.

**Methods:**

The study consists of two parts: modeling and validation. In modeling, patients scheduled for esophagogastroduodenoscopy (EGD) or colonoscopy sedation are enrolled. The modified observer’s assessment of alertness/sedation (MOAA/S) score < 4 is defined as loss of response to represent moderate sedation. Two patient groups receiving bronchoscopy or endoscopic retrograde cholangiopancreatography (ERCP) are used for validation. Model performance is assessed by receiver operating characteristic (ROC) curves and area under the curve (AUC). Simulations are performed to demonstrate how the model is used to rationally determine drug regimen for moderate sedation.

**Results:**

Interaction between propofol and alfentanil is stronger than the other pairwise combinations. Additional synergy is observed with three drugs. ROC AUC is 0.83 for the modeling group, and 0.96 and 0.93 for ERCP and bronchoscopy groups respectively. Model simulation suggests that 1 mg midazolam, 250 µg alfentanil and propofol maximally benefits from drug interactions and suitable for moderate sedation.

**Conclusion:**

We demonstrate the accurate prediction of a three-drug response surface model for moderate sedation and simulation suggests a rational dosing strategy for moderate sedation with midazolam, alfentanil and propofol.

**Supplementary Information:**

The online version contains supplementary material available at 10.1186/s40360-023-00642-5.

## Introduction

The number of noninvasive or minimally invasive gastrointestinal endoscopies has grown over the past decades. Procedural sedation is needed for many of these procedures. The key elements in sedation are patient safety and comfort. Concerns for safety parallels greater patient comfort, which is achieved with deeper anesthesia and more likely to curb respiratory function. A motionless patient is not required during the entire procedure and some patient movement is allowed if it does not interfere with ongoing procedures [1]. To balance this, sedation can be lightened.

Sedation is a continuum and large individual variations exist. This variation leads to unwanted deep sedation and respiratory depression that follows it. It is therefore reasonable to aim for moderate sedation in selected patients or procedures as opposed to heavy sedation [2–4]. Regulations or statements have granted non-anesthesiology physicians who are qualified by education, training and licensure the permission to conduct moderate sedation [5]. High level of patient satisfaction is reported for ambulatory procedures under moderate sedation [6], and recall during light to moderate sedation is infrequent [7–9].

Drug interaction is fundamental in monitored anesthesia care. Insights regarding anesthetic drug interactions have advanced considerably in the last decades [10]. We are able to visualize and estimate most combinational drug effects with the help of response surface models [11]. Information such as the degree of interaction and dose-response curve shifts can easily be extracted from a single response surface, and it can be used to predict patient response [12]. While most models work with two drugs, many sedation regimens contain three drugs. We adopt one of the three-drug models to enhance the model’s utility in multiple clinical scenarios [3, 13].

The concern of over-sedation, given by inexperienced healthcare providers, calls for an educational training model. Typical milligram per body weight drug dosing is very unreliable and does not take drug interactions into account, which are usually synergistic in both desirable and undesirable effects [14]. Here we describe the development and validation of a three-drug response surface pharmacodynamics model [3] that accommodates drug interactions suitable for moderate sedation during procedural sedation.

## Methods

The study consisted of two sections. First part describes the development of a response surface model from a published patient population undergoing gastrointestinal endoscopy [3]. We reprocessed the data to handle Modified Observer’s Assessment of Alertness/Sedation [15] (MOAA/S, Table 1) scale < 4 rather than < 2 to distinguish between moderate and heavy sedation. In brief, two assessors trained in MOAA/S scored sedation at the start and the end of the examination, or at critical events (endoscope insertion, painful expression, return of consciousness) for each study patient. Adverse events related to anesthesia, or the endoscopy procedures were recorded.

**Table 1:** Modified Observer’s Assessment/Alertness Sedation scale [15]

Observation	Score
Responds readily to name spoken in normal tone	5
Lethargic response to name spoken in normal tone	4
Responds only after name is called loudly and/or repeatedly	3
Responds only after mild prodding or shaking	2
Does not respond to mild prodding or shaking, but responds to noxious stimuli	1
Does not respond to noxious stimuli	0

Loss of response for moderate sedation was defined as a score < 4

The second part validates the moderate sedation model in two procedures: bronchoscopy and endoscopic retrograde cholangiopancreatography (ERCP). Stimuli in ERCP resembles gastrointestinal endoscopy and bronchoscopy represents a more noxious procedure. The study was approved in line with the principles of the declaration of Helsinki, and approval was granted by the Institutional Review Board of Taipei Veterans General Hospital (IRB 2019-01-007BC and 2021-07-002BE).

### Study group (modeling)

 Details of the patients and sedation management for the modeling group were described in an earlier publication [3]. In brief, ASA class I or II adult patients (20 to 80 years of age) undergoing gastrointestinal endoscopy sedation were enrolled. Sedation was performed with propofol, midazolam and alfentanil in every patient.

### Study group (Validation)

Patients qualified as ASA Class I to III, aged 20 to 80 years scheduled for ERCP and bronchoscopy were enrolled. All written informed consents were obtained after thorough discussion with the participants. Drugs were given through a 22-gauge i.v. catheter placed in a distal arm. Patients were monitored using standard monitored anesthesia care equipment: electrocardiography, pulse oximetry, and non-invasive blood pressure. Supplemental oxygen was given by nasal cannula at 3 to 5 L/min. Drug regimen was not restricted and determined by the anesthesiologist in charge. All sedation drugs were selected from propofol, midazolam or alfentanil. Drug doses and timing were recorded. After each bolus, the medication was flushed with 3 mL of normal saline.

The MOAA/S score was used to measure sedation level from clinical observation by the attending anesthesiologist familiar with the scoring on a 0 to 5 scale, where 5 was awake and 0 was unresponsive to noxious stimuli. Loss of response (LOR) was defined as MOAA/S < 4, indicating moderate sedation. All procedures were performed by experienced specialists. Critical moments that were considered crucial for model development were recorded, which included the induction phase transition from wakefulness to LOR, every instrumentation or procedural maneuver to obtain specimen or treatment, and emergence transition back to wakefulness. Other MOAA/S recordings were not restricted if the attending anesthesiologists wished to obtain.

### Pharmacodynamic response surface modeling

 The nonlinear mixed amount with zero amount (NLMAZ) model was used [3, 16]. Patient response was reprocessed into binary data (1 if MOAA/S is 0 to 3, and 0 if MOAA/S is 4 to 5) for modeling. The formula is as follows.

1 $$\text{E}=\frac{{\left(\frac{U}{{U}_{50}}\right)}^{n}}{1+{\left(\frac{U}{{U}_{50}}\right)}^{n}}$$

Where E is the effect, defined as the probability of LOR. U_50_ is the value of U to achieve 50% chance of LOR, of half maximal effect. U resembles that in the Minto model [17], which can be interpreted as a new drug and is the sum of the normalized potency of midazolam, alfentanil and propofol (Eqs. 2 and 3):

2 $${\text{U}}_{m}=\frac{{C}_{m}}{{C}_{50 m}};{ \text{U}}_{a}=\frac{{C}_{a}}{{C}_{50a}}; { \text{U}}_{p}=\frac{{C}_{p}}{{C}_{50p}}$$

3 $$U={\text{U}}_{m}+{{\text{U}}_{a}+\text{U}}_{p}$$

The variables C_m_, C_a_ and C_p_ refer to the calculated Ce of midazolam, alfentanil, and propofol respectively. The subscripts m, a, p will refer as midazolam, alfentanil and propofol respectively throughout the article. C_50_ is defined as the concentration of drug required to elicit half maximal effect. It is the same concept with U_50_. For the model to scale correctly, we have to define:

4 $$1=\text{x}+\text{y}+\text{z}=\frac{{\text{U}}_{m}}{U}+\frac{{\text{U}}_{a}}{U}+\frac{{\text{U}}_{p}}{U}$$

5 $$\text{x}=\frac{{\text{U}}_{m}}{U}; \text{y}=\frac{{\text{U}}_{a}}{U}; \text{z}=\frac{{\text{U}}_{p}}{U}$$

Where x, y and z are the fractions of midazolam, alfentanil and propofol for any given observation. All the unknown parameters are expressed with the full cubic form of the canonical polynomial in Eq. 6:

6 $$\text{P}=\sum {\alpha }_{i}{x}_{i}+\sum {\beta }_{ij}{x}_{i}{x}_{j}+\sum {\gamma }_{ij}{x}_{i}{x}_{j}\left({x}_{i}-{x}_{j}\right)+\sum {\delta }_{ijk}{x}_{i}{x}_{j}{x}_{k}$$

The Greek letter constants are the vector constants. Each is responsible for the respective single, pairwise or triple drug interaction. The parameters U and n (Eq. 1) both possess individual vector constants. In total, there will be 20 vector constants and 3 C_50_ parameters that need to be estimated from model fitting.

Pharmacokinetic profiles for effect-site drug concentrations (Ce) were calculated using a simulation program (TIVA trainer-Version 9.1, Build 5, Euro SIVA). The Maitre model [18] was used for alfentanil, Zomorodi model [19] for midazolam and Schnider model for propofol. The t_1/2_ k_e0_ values in the program were from EEG analyses by Scott et al. [20] for alfentanil and Buhrer [21] et al. for midazolam. TIVA trainer allowed users to simulate anesthetic drug Cp (plasma concentration) and Ce by inputting patient demographics. The models in the TIVA trainer program have been extensively tested and validated against real-world data. The TIVA trainer program is similar to the Target Controlled Infusion (TCI) pumps used in routine anesthesia, with parameters and concentration levels derived in the same way.

The model was fit to propofol, alfentanil, midazolam Ce and in the presence or absence of LOR during endoscopy procedures. Model parameters were estimated with Matlab software (R2021b, The MathWorks, Inc., Natick, MA). Matlab’s built-in function, *fmincon()*, was selected, and an iterative process (2000 iterations) utilizing the bootstrap method [3, 14] was used to find the local minima of -2 times the logarithm of the maximum likelihood (-2LL) in Eq. 7.

7 $$-2\text{L}\text{L}=-2\times {\sum }_{i=1}^{K}\left[Ri\times ln\left(Pi\right)+(1-Ri)\times ln(1-Pi)\right]$$

K was the number of pooled observations. Ri is the response to stimuli measured by MOAA/S (Ri = 0 for score 0 to 3, Ri = 1 for score 4 or higher). Pi, the probability for LOR to stimuli was calculated from the model. The relative standard errors (RSE) of the model parameters were calculated by dividing the standard error by the estimated parameter value.

Accuracy was defined as an absolute difference less than 0.5 between the binary patient responses and the predicted probability of LOR. The discriminating power of the model in both patient groups was measured using receiver operating characteristic (ROC) curves. ROC curve input was the observed and model predicted responses in both patient groups. Area under the curves (AUCs) and 95% confidence interval were calculated to objectively assess the performance of the RSM.

### Pharmacodynamic response surface model validation

Data from the bronchoscopy and ERCP patient groups were validated. To fully validate our model, the original MOAA/S < 2 model and the MOAA/S < 4 model from this study were both assessed. Drug Ces were fed to the model and the predicted probabilities of LOR were calculated. The prediction was compared to the observed patient MOAA/S. Accuracy was defined the same as the modeling conditions.

### Pharmacodynamic response surface model simulation

A simplified simulation illustrates how the model could guide clinical dosing as an advisory system. A comprehensive simulation was not possible due to model complexity. It was common to target for model predicted 95% chance of LOR in simulations to ensure most patients reached LOR [22]. Three regimen categories were demonstrated.

1. Two-drug regimen: propofol alone, propofol with 1 mg midazolam and propofol with 2 mg midazolam.
2. Two-drug regimen: propofol alone, propofol with 250 µg alfentanil, and propofol with 500 µg alfentanil.
3. Three-drug regimen: propofol with 250 µg alfentanil and 1 mg midazolam.

The simulation will demonstrate the reduction of propofol concentration required to achieve 95% LOR, a phenomenon called curve-shifting.

## Results

### Patient characteristics and pharmacokinetic data

The modeling group had 56 patients with a total of 227 observations. 59% of the patients were female. Mean (standard deviation) age and BMI were 53.3 (13.3) years and 23.1 (3.7) kg/m^2^. Drug concentration ranged from 0 to 108 ng/mL for midazolam, 0–156 ng/mL for alfentanil and 0–2.6 µg/mL for propofol.

For validation, there were 20 patients in each of the bronchoscopy and ERCP group. The demographics are listed in Table 2. There were 723 and 171 observations in the ERCP and bronchoscopy group respectively. MOAA/S recordings during ERCP were more abundant because assessments were performed by different anesthesiologists who recorded more frequently during the procedure. Drug concentration ranged from 0 to 53 ng/mL for midazolam, 0–62 ng/mL for alfentanil and 0–3.5 µg/mL for propofol for the bronchoscopy group. All patients received the three drugs during the procedure.

**Table 2:** Patient demographics for the validation groups

	Bronchoscopy	ERCP
n	20	20
Age (SD)	60.5 ± 11.3	58.9 ± 11.4
F/M (F %)	11/9 (55%)	11/9 (55%)
BMI (SD)	23.3 ± 3.1	24.4 ± 3.0
Sedation Time (min)(SD)	41.15 ± 14.3	38.8 ± 9.8

*ERCP* Endoscopic retrograde cholangiopancreatography. *BMI *Body mass index (kg/cm^2^)

Drug range for the bronchoscopy group was 0–47 ng/mL for midazolam, 0–23 ng/mL for alfentanil and 0–3 µg/mL for propofol. Seven patients received propofol and alfentanil without midazolam, and the rest received all three medications during the procedures. Patients in both groups completed the examinations smoothly and uneventfully.

### Pharmacodynamic response surface model

The model parameters were listed in Table 3. C_50,mid_ and C_50,prop_ were below the maximal drug concentration used for modeling, but C_50,alf_ was not. A large C_50,alf_ indicated alfentanil was incapable of eliciting LOR alone.

**Table 3:** Model (MOAA/S < 4) parameters derived from gastrointestinal endoscopies

Parameter	Estimate	Parameter	Estimate
**C**_**50,alf**_	340.84	**δ**_**U,map**_	-0.65
**C**_**50,mid**_	40.73	**α**_**n,m**_	3.95
**C**_**50,prop**_	1.21	**α**_**n,a**_	13.40
**α**_**U,m**_	-0.34	**α**_**n,p**_	10.66
**α**_**U,a**_	-0.65	**β**_**n,ma**_	0.00^a^
**α**_**U,p**_	-0.50	**β**_**n,mp**_	0.00^a^
**β**_**U,ma**_	-0.65	**β**_**n,ap**_	0.00^a^
**β**_**U,mp**_	-0.40	**γ**_**n,ma**_	0.00^a^
**β**_**U,ap**_	-0.75	**γ**_**n,mp**_	0.00^a^
**γ**_**U,ma**_	-0.04	**γ**_**n,ap**_	0.00^a^
**γ**_**U,mp**_	-0.04	**δ**_**n,map**_	0.00^a^
**γ**_**U,ap**_	-0.04		

^a^The values are not zero but rounded to zero due to assigned values ranged in the magnitude of 10^− 4^ to 10^− 7^

**Fig. 1:**
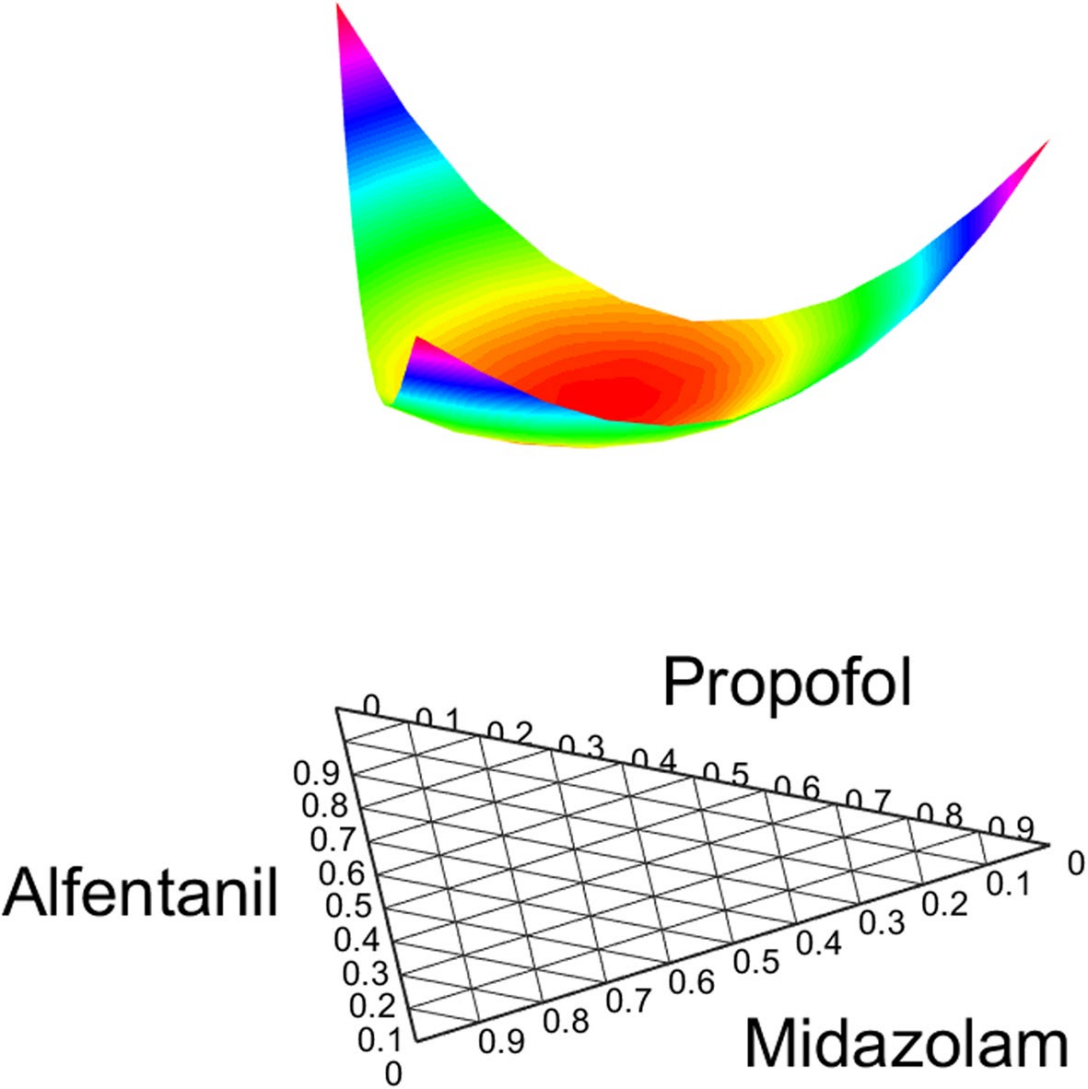
Ternary plot of the parameter U_50_. Downward bowing of the surface indicates synergistic effects between the pairwise combinations

The final response surface was a 4-axes graph (3 drug axes and 1 effect axis) that cannot be visualized directly. Alternatively, the ternary plot (Fig. 1) was used to illustrate drug interactions by graphing U_50_. U_50_ is conceptually a new drug formed by combinations of the three drugs. Mathematically, vector constants for U_50_ controlled the type of interaction between the drugs. Any synergistic interaction would lower U_50_. Negative values decreased U_50_ and suggested synergism. β_U,ap_ was − 0.75, which was indicative of stronger interaction between propofol and alfentanil than the other pairwise combinations. This was followed by midazolam-alfentanil (β_U,ma_ = -0.65), then midazolam-propofol (β_U,mp_ = -0.4).

The triple interaction term δ_U,map_ was − 0.65, which indicated modest additional synergism when all three drugs are given. Minimal value of U_50_ was 0.61 (thus the point of strongest drug interaction), occurring at equal fraction of the three drugs. For comparison, the nadirs of U_50_ for the pairwise combinations were 0.64 for propofol-alfentanil, 0.74 for midazolam-propofol, and 0.69 for midazolam-alfentanil, in which all occurred at equal drug fractions.

### Modeling and validation performance

**Fig. 2:**
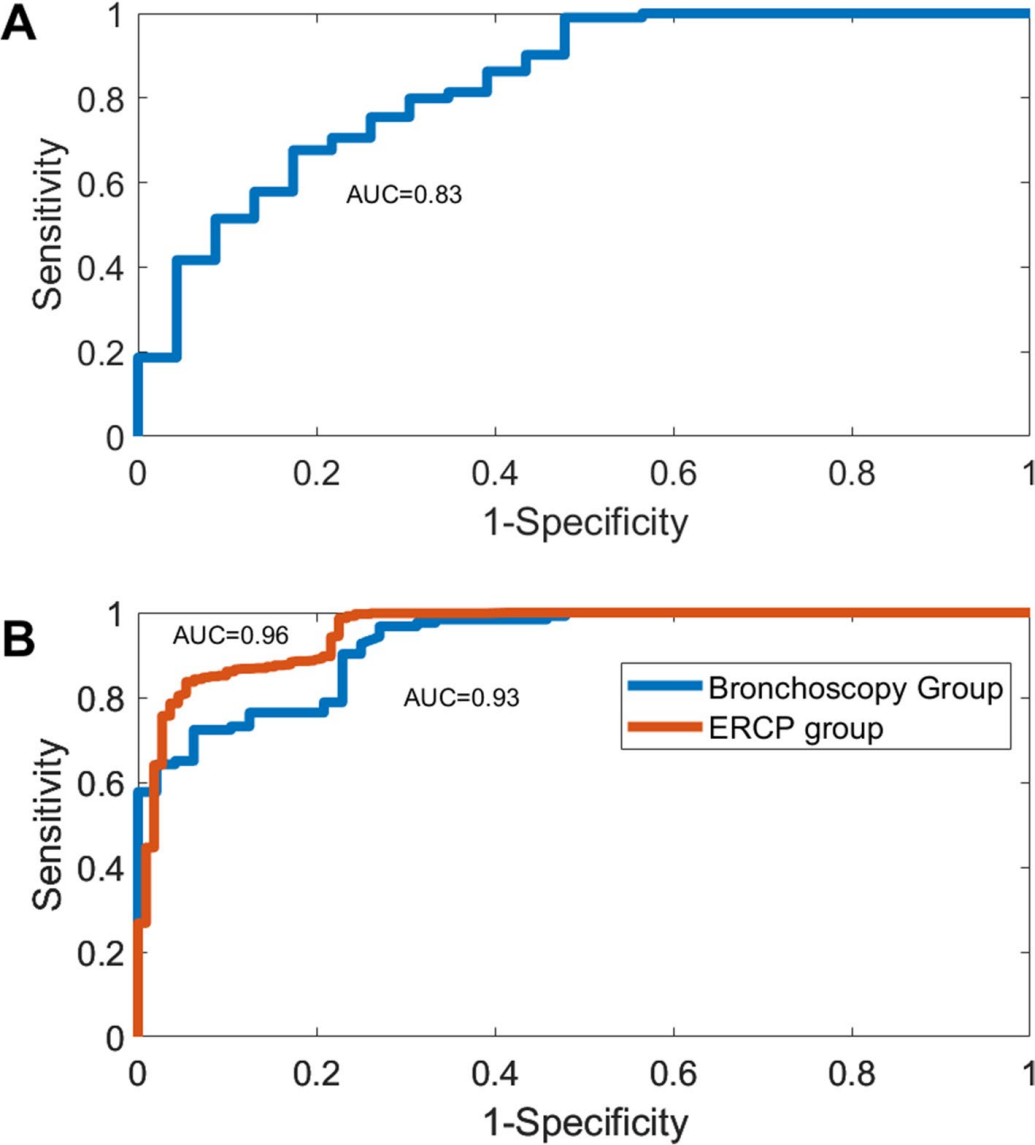
ROC curve of the MOAA/S < 4 model and validation groups. Panel **A** ROC of the modeling group. Panel **B** ROC of the validation groups. AUC = Area Under the Curve; ERCP = endoscopic retrograde cholangiopancreatography; MOAA/S = Modified Observer’s Assessment of Alertness/Sedation; ROC = Receiver’s Operating Characteristics

The MOAA/S < 4 model had an internal predefined accuracy of 93.4%. The ROC AUC was 0.83 (Fig. 2A).

MOAA/S < 4 model prediction accuracy was 94.2% (ROC AUC 0.96) and 88.9% (ROC AUC 0.93) for the ERCP and bronchoscopy group respectively (Fig. 2B). The accuracy of the published MOAA/S < 2 model was 86.5% in ERCP and 92.4% in bronchoscopy group. This implied good prediction made by the model for both moderate and heavy sedation. Accuracy of model prediction in the 7 patients in the bronchoscopy group was 69%.

### Model simulation

**Fig. 3:**
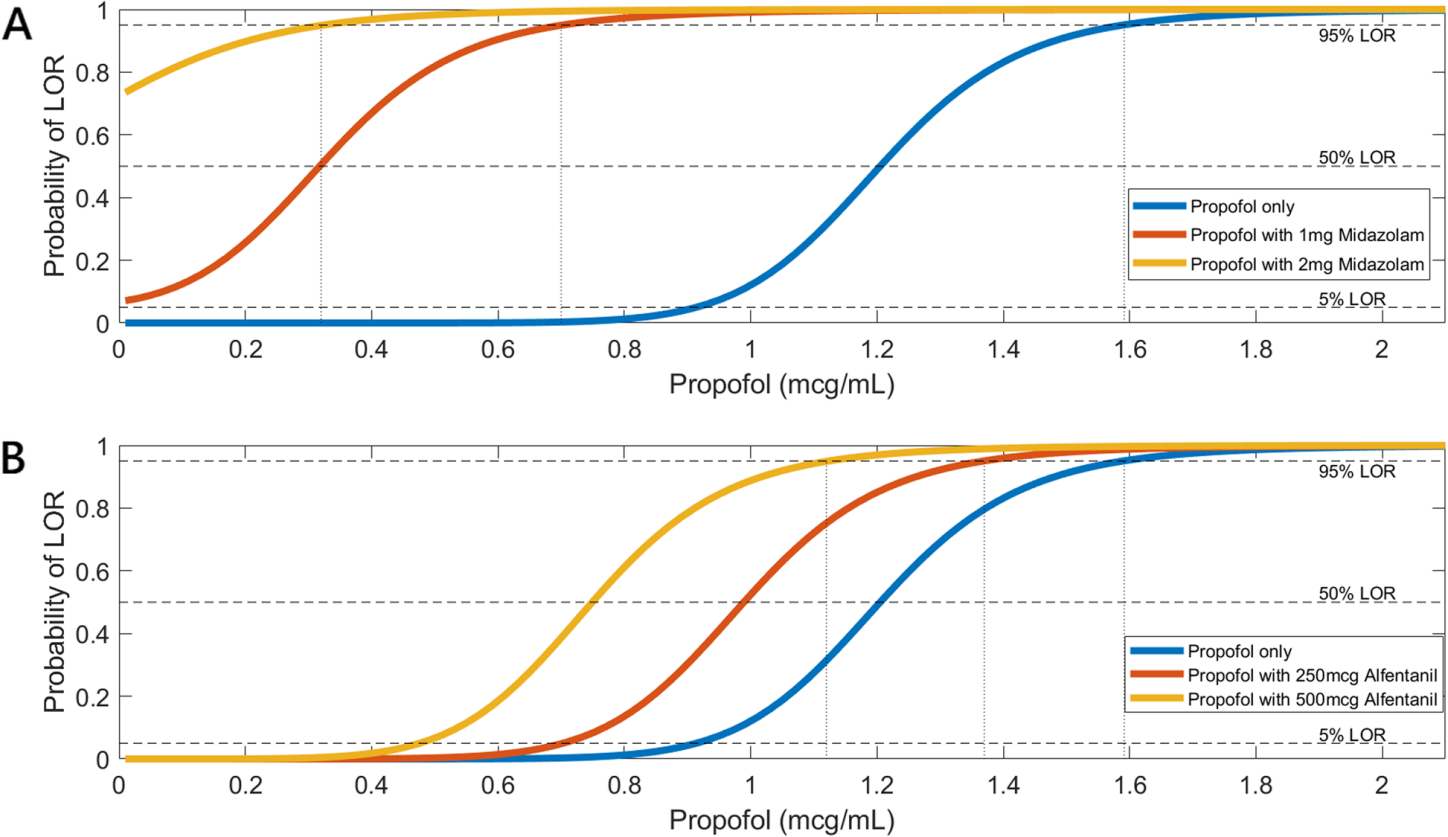
Curve shift of Propofol with midazolam or alfentanil. Panel **A** Sedation without alfentanil. The dashed lines represent 95%,50% and 5% chance of LOR. The propofol concentration, when given alone, to achieve 95% chance of LOR is 1.59 μg/mL. This requirement is lowered to 0.7 and 0.32 μg/mL if coupled with midazolam 1 mg and 2 mg respectively (dotted lines). Panel **B** Sedation without midazolam. Propofol concentration is 1.59, 1.37 and 1.12 μg/mL when given alone, with 500 μg and 250 μg alfentanil

Figure 3 showed the curve shift effects of propofol without alfentanil (Panel A) and propofol without midazolam (Panel B). Midazolam and alfentanil were simulated as a bolus and their maximal Ce were used. After a bolus of 1 mg and 2 mg midazolam, the maximal Ce were 21 and 52 ng/mL respectively. Maximum alfentanil Ce were 27 and 55 ng/mL after a bolus of 250 and 500 µg respectively. The dashed lines represent 95%, 50% and 5% chance of LOR. Simulations often adopt the 5% LOR line as return of consciousness and 95% LOR line as the LOR threshold [22, 23]. The propofol concentration, when given alone, to achieve 95% chance of LOR is 1.59 µg/mL. This requirement is lowered to 0.7 and 0.32 µg/mL if coupled with midazolam 1 mg and 2 mg respectively (dotted lines). Propofol concentration is 1.59, 1.37 and 1.12 µg/mL when given alone, with 500 µg and 250 µg alfentanil to achieve 95% chance of LOR.

**Fig. 4:**
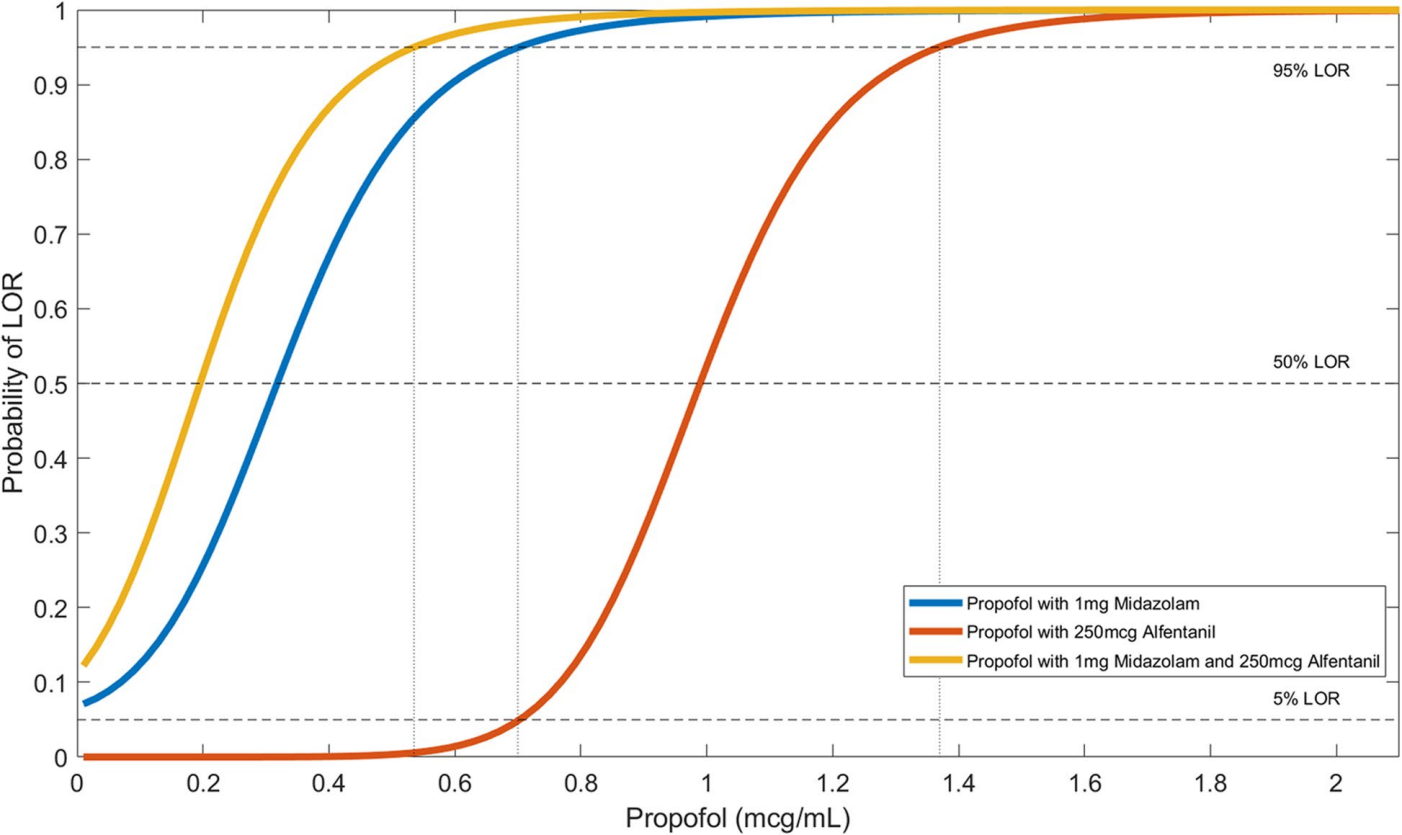
Curve shift of Propofol with midazolam and alfentanil

The three-drug regimen was graphed in Fig. 4. It was overlayed with other two-drug regimens (propofol + 1 mg midazolam, and propofol + 250 µg alfentanil) for comparison. The propofol concentration required to achieve 95% LOR was further lowered to 0.54 µg/mL in the three-drug regimen. The three-drug regimen lowered propofol requirements greater than the pairwise combinations. The synergistic benefit supported the rationale to continue to use the regimen of 1 mg midazolam, 250 µg alfentanil and propofol.

## Discussion

We demonstrated good prediction accuracy of the NLMAZ model for moderate procedural sedation. The MOAA/S < 4 model for moderate sedation had a prediction accuracy of at least 88% during bronchoscopy and ERCP.

We focused on the drug effect interactions in this study. In short procedures such as gastrointestinal endoscopy, drug metabolism or elimination was negligible. Distribution of the drugs among the different body compartments became the deciding factor to elicit drug effects. The simulation program TIVA trainer and the commercial pumps in routine anesthesia used the same pharmacokinetic parameters. These drug models had been extensively studied and validated [24–27]. Drug distribution was thus reliable using the simulation program.

Patient satisfaction became a concern when physicians are prompted to reduce sedation levels. Intuitively patients expected to be completely unresponsive during the examinations. However, it did not always translate into higher satisfaction. Several studies showed that moderate sedation could achieve good patient and operator satisfaction, even with some patient movement and recall. VaNatta et al [28] reported moderate sedation with a combination of fentanyl, propofol or midazolam reached good patient satisfaction in the recovery unit, a score that was not significantly different from that of heavy sedation with propofol alone. Heavy sedation was defined as MOAA/S < 2, which was consistent with our definition. A meta-analysis reported patient satisfaction greater than 88% and operator satisfaction greater than 85% in gastrointestinal procedures under moderate sedation [29]. Another study aimed at moderate sedation had a mean MOAA/S score of 3 reported good patient satisfaction of 9 ~ 10 out of a scale of 10 [30].

Sedation was a continuum without clear boundaries between stages. Unexpected deep sedation was common even when drugs dosages were within guidelines [31]. Literatures have described moderate sedation using a variety of opioids and sedatives. Many unwanted events still occurred, especially when different medications were given together [31], commonly an opioid and a sedative [29]. Drug pharmacokinetics and their interactions were often neglected and only the total dosages were recorded in most studies comparing different sedation regimens. Adverse events also exhibited synergism. Typical tools that were used for interaction studies included the isobolograms and curve-shift effects. Response surface models were the fusion of the two and illustrated a more complete overview of the entire interaction spectrum across a wide range of drug concentrations.

Interactions between drugs could be classified into additive, synergistic or infra-additive [12]. Most anesthetics exhibited synergistic behavior among different classes, and are only modestly synergistic or additive with the same class. Midazolam and propofol were considered hypnotics where the primary site of action were cerebral GABA receptors. The pairwise interaction parameter in the NLMAZ model were β_U,ap_, β_U,ma_ and β_U,mp_. The interaction of midazolam-propofol was the weakest of the three (β_U,mp_= -0.4), which echoed the result of an earlier triple-drug interaction study [3]. It was a reasonable finding since an analgesic was more suitable to alleviate noxious stimuli. Synergism was a powerful feature that allowed physicians to achieve the same outcome with lower doses.

A large C_50,alf_ was not achievable in practice. This implied the inability of opioids to produce reliable sedation alone [14, 32]. It was still not achievable even with drug-drug synergism. The greatest synergy given by the model was at equal drug fractions. A fraction of 0.33 for alfentanil was far greater than the suggested cut-off (0.12) to avoid respiratory depression [3].

It was common for a model to mathematically extrapolate effect predictions beyond the original concentration ranges. The extrapolation, however, would have doubtful accuracy. Midazolam and alfentanil concentrations in the validation group were within the drug concentrations for modeling, but propofol concentrations were partly outside of it. The resulting predictions were reasonably accurate.

A three-drug model must be able to downscale to accurately predict single or drug pairs. There were seven patients who received only propofol and alfentanil, in which 69% of the predictions were accurate. A drop in accuracy was anticipated but still acceptable when working with categorical data. The results had two important implications:

1. Validated the extrapolated model prediction for propofol outside the modeling condition, and.
2. Examined the three-drug model’s accuracy to downscale to two-drug regimens.

Figure 4 illustrated how drastically the required drug concentrations to produce 95% LOR could be reduced in the presence of a second drug. Propofol concentration at 95% LOR was 1.59 µg/mL, which was close to the manufacturer setting of wake-up concentration 1.5 µg/mL. One study inspected closely on the wake-up concentration (C_50_) of propofol combined with fentanyl (1 ng/mL) during short term infusion [33]. The wake-up concentration of propofol was 1.0 µg/mL, which was close to our 50% LOR concentration (0.96 µg/mL) of propofol combined with 250 µg alfentanil (Fig. 3B). Fentanyl potency was estimated 16 to 70 times than that of alfentanil [34–37], and our simulated alfentanil concentration was within that range for comparison.

Our study had several limitations. First, our data was clustered and did not include extreme concentrations. The use of patients instead of volunteers limited our ability to obtain equally distributed data including the extreme ranges of administered drugs. Large doses were not in the protocol and would not be acceptable. Extreme drug dosing would perfect the model but unnecessary in practice.

Second, our data did not reach steady state drug concentrations. Bolus dosing rarely did and was common in procedural sedation. We believed the unfavorable modeling condition better reflected practice.

## Conclusion

We demonstrated the ability to accurately predict the effects of combining different anesthetic drugs using a triple-drug response surface model. Dose reduction was mandatory when combining different anesthetic drugs as implied by the model. This is an important consideration for trainees, who must be taught to consider drug interactions when administering anesthesia. Our model provides a visual representation of drug interactions, allowing users to better understand how to adjust anesthetic dosing in different situations.

## Supplementary Information

**Additional file 1: Table S1.** Raw data for the modeling group.

**Additional file 2: Table S2.** Raw data for the validation group.

## Data Availability

The datasets generated and/or analysed during the current study are included in this published article as supplementary information file.
